# Trajectories of emotional and behavioral problems from childhood to early adult life

**DOI:** 10.1177/1362361320908972

**Published:** 2020-03-19

**Authors:** Dominic Stringer, Rachel Kent, Jackie Briskman, Steve Lukito, Tony Charman, Gillian Baird, Catherine Lord, Andrew Pickles, Emily Simonoff

**Affiliations:** 1 King’s College London, UK; 2 South London and Maudsley NHS Foundation Trust, UK; 3 Guy’s and St Thomas’ NHS Foundation Trust, UK; 4 UCLA Semel Institute for Neuroscience and Human Behavior, USA

**Keywords:** autism, emotional and behavioral problems, longitudinal, mental disorders, Strengths and Difficulties Questionnaire

## Abstract

Little is known about their stability and the factors that influence their persistence or change over the life-course. To address this, we use data from 158 participants from the Special Needs and Autism Project cohort studied at three time-points from 12 to 23 years. We used latent growth models to study the role of child, family, and contextual characteristics on the conduct, emotional, and attention deficit hyperactivity disorder domains of the parent-reported Strengths and Difficulties Questionnaire. Symptoms decreased significantly over time for all three domains, but many participants still remained above the published cutoffs for likely disorder on at least one of the three domains. Individual trajectories showed high levels of persistence. Higher initial adaptive function and language levels predicted a greater decline in conduct and attention deficit hyperactivity disorder symptoms. In contrast, increased emotional symptoms were predicted by higher language functioning, lower levels of autism symptom severity and higher parental education. Greater neighborhood deprivation was associated with more conduct problems but also a greater decline over time. Our findings highlight that it may be possible to accurately predict mental health trajectories over this time period, which could help parents and carers in planning and help professionals target resources more efficiently.

**Lay Abstract:**

Although mental health problems are common in autism, relatively little is known about their stability and the factors that influence their persistence or change over the life-course. To address this, we use data from the Special Needs and Autism Project (SNAP) cohort studied at three time-points from 12 to 23 years. Using the parent-reported Strengths and Difficulties Questionnaire (SDQ) domains of conduct, emotional, and ADHD symptoms, we evaluated the role of child, family, and contextual characteristics on these three trajectories. Symptoms decreased significantly over time for all three domains, but many participants still scored above the published disorder cutoffs. Individuals showed high levels of persistence. Higher initial adaptive function and language levels predicted a greater decline in conduct and ADHD symptoms. In contrast, higher language functioning was associated with higher levels of emotional symptoms, as was lower levels of autism symptom severity and higher parental education. Those with higher neighborhood deprivation had higher initial conduct problems but a steeper decline over time. Our findings highlight that it may be possible to accurately predict mental health trajectories over this time period, which could help parents and carers in planning and help professionals target resources more efficiently.

## Introduction

In the past decade of autism research, there has been increasing appreciation of the high rates and impact of additional psychiatric problems and disorders among people with autism spectrum disorders. These elevated rates are present in both clinical and population-based samples and are observed across the lifespan from early childhood to adult life (Lai et al., 2019). In considering the prevalence of psychopathology, it is important to focus wherever possible on population-based studies as the potential confounders in clinically ascertained studies make the results difficult to interpret. Studies in preschool and primary school-aged children suggest overall rates of symptoms and impairment meeting diagnostic criteria for additional disorders in 90% of 4 to 9-year-old children with autism from a community sample (Salazar et al., 2015); this contrasts with rates of 12%–16% in separate typically developing populations assessed with the same instrument (Egger & Angold, 2006; Wichstrom et al., 2012). Anxiety disorders were particularly prominent, occurring in 79%, but attention deficit hyperactivity disorder (ADHD) and oppositional defiant disorder (ODD) were also common, present in 59% and 29% of autistic children, respectively. Using the Strengths and Difficulties Questionnaire (SDQ; (Goodman, 1997)) with binary cutoffs to examine mental health problems in the domains of emotional problems, behavioral difficulties and ADHD, the UK Millennium Cohort Study reported that 5-year-old children with autism spectrum disorder (ASD), without or with intellectual disability (ID), had increased risk of disorder, with odds ratios and observed prevalence of 2.6 for conduct (46% without ID, 58% with ID), 5.7 for emotional (38%, 39%) and 6.8 for ADHD (59%, 88%) (Totsika, Hastings, Emerson, Berridge, & Lancaster, 2011).

In middle childhood and adolescence, studies using population-based (Simonoff et al., 2008), special school (Gjevik et al., 2011) and intellectually “higher functioning” (Mattila et al., 2010) samples all report high rates of psychopathology, with overall rates of additional psychiatric disorders in about 70%. Rates of individual disorder groups vary with anxiety disorders occurring in 14%–42%, ADHD in 28%–31% and ODD in 4%–28% remaining the most common diagnoses (Gjevik et al., 2011; Simonoff et al., 2008). As with younger children, using SDQ cutoffs, the adjusted odds ratios for mental health problems were substantially increased over the general population comparator: 4.1 (conduct: 64% without ID, 65% with ID), 8.3 (emotional: 74%, 71%) and 10.3 (ADHD: 85%, 87%) (Totsika, Hastings, Emerson, Lancaster, & Berridge, 2011).

In adult life, population-based studies applying rigorous diagnoses of autism and co-occurring conditions are not currently available. Data from the Scottish national census comparing those with self-reported autism to the rest of the population found 38% versus 5.7% reported an additional mental health condition (Rydzewska et al., 2018). Among adults with an ASD diagnosis in a large, integrated health system in a US primary care organization, 54% had an additional psychiatric diagnosis, with the increased risk of diagnosis greatest for psychotic disorders and obsessive compulsive disorder, and with anxiety and depressive disorders increased in the region of 5- to 6-fold (Croen et al., 2015). A Dutch cohort ascertaining adults with ASD from a wide range of sources but including only those with IQ > 80 identified at least one psychiatric disorder in 79% versus 49% of their comparison non-ASD population, with mood (57%) and anxiety disorders (54%) the most common (Lever & Geurts, 2016). Many adult studies of psychiatric disorders have not included ADHD or other behavioral conditions such as ODD or antisocial personality.

Hence, while absolute rates vary across studies, reflecting differences in samples and measurement, where comparison rates are provided, those with autism are consistently higher. Furthermore, studies across the age range report high rates of multiple co-occurring conditions, including across the domains of emotional and behavioral disorders. In the early childhood period, Salazar et al. (2015) reported that nearly 80% of the sample met criteria for more than one diagnosis, while in middle childhood Simonoff et al. (2008) reported 71% with multiple psychiatric disorders using parent-reported psychiatric interviews.

Although rates of mental health problems and disorders in autism are high at every age studied, relatively few studies have examined the patterns of stability and change over time and the factors that influence these. The absence of a body of research likely reflects its relative novelty. In a previous longitudinal analysis of the population-based Special Needs and Autism Project (SNAP) cohort, we showed that there was considerable stability over a 4-year period within the domains of conduct, emotional and ADHD symptoms and, further, that the domain specificity seen in parent reports remained when correlating teacher reports at age 12 years with parent reports at 16 years (Simonoff et al., 2013). Among the longitudinal studies in autism examining the course of mental health and related problems from childhood/adolescence to adult life, three studies report an overall decline in observable and maladaptive symptoms, arguably overlapping with behavioral problems measured in non-autistic populations (Anderson et al., 2011; Gray et al., 2012; Shattuck et al., 2007). In contrast, the fourth, the Early Diagnosis of ASD (EDX) study, exploring trajectories of anxiety and depression found increased symptoms from age 13 to 24 years (Gotham et al., 2015).

The present study uses data from the SNAP cohort at three timepoints over 11 years from middle childhood to early adult life to explore the trajectories of mental health symptoms, focusing on emotional, behavioral and ADHD symptoms. Using latent growth curve modeling, we asked the following questions. First, do symptom scores change over this time period? Second, can we identify factors present in middle childhood that predict the symptom scores over time? Finally, we wished to identify characteristics present in childhood that predict differences in symptom trajectories.

## Method

### Participants

All members of the SNAP cohort who received an International Statistical Classification of Diseases and Related Health Problems-10 (ICD-10) diagnosis of any pervasive developmental disorder (PDD), including childhood autism, Asperger Syndrome, PDD—not otherwise specified or atypical autism at the first wave of data collection were eligible for participation. These diagnoses are now all subsumed under the *Diagnostic and Statistical Manual of Mental Disorders* (5th ed.; *DSM*-5) category of autism spectrum disorder (ASD) and the term “autism” is used subsequently in this paper to refer to this cohort. The SNAP sample was originally drawn from a total population cohort of 56,946 children born between July 1990 and December 1991 in 12 districts in south-east England, described in detail elsewhere (Baird et al., 2006). All those with a clinical diagnosis of any PDD (*N* = 255) or considered “at risk” for being an undetected case by virtue of having a statement of Special Educational Needs (SEN; *N* = 1,515) were surveyed using the Social Communication Questionnaire (SCQ (Rutter et al., 2003)). A total of 255 children aged 10–12 years (*wave 1*) were assessed for an ASD using the Autism Diagnostic Interview–Revised (ADI-R; (Lord et al., 1994)), Autism Diagnostic Observation Schedule–Generic (ADOS-G; (Lord et al., 2000)) as well as an evaluation of language and intellectual and adaptive functioning. Of these, 158 (132 male, 16 female) met criteria for an ASD (81 childhood autism, 77 other PDDs). A subsample of 100 youth with autism and IQ > 50 participated at age 15–16 (*wave 2*), a study to explore the cognitive phenotype of autism. At age 23 (*wave 3*), we attempted to contact the families of all 158 autistic participants and successfully completed assessments on 126 (80%; 110 male), who represent the denominator for all descriptive statistics given below (Supplementary Figure 1).

Ethical approval for the most recent data collection wave was given by the Camberwell and St. Giles NRES Committee number 12/LO/1770, IRAS project number 112286. Written informed consent was obtained from all participating parents and all autistic adults who had the mental capacity to give consent. Where researchers judged that an autistic adult did not have the capacity to consent, a consultee was appointed to determine whether the young adult would wish to participate if he or she had been able to give informed consent.

### Measures

#### Outcome variables

The parent-reported SDQ (Goodman et al., 2000) measured current *co-occurring mental health symptoms* broken down into the conduct, emotional and ADHD subscales. The SDQ is widely used as a screening instrument for child mental health problems and its psychometric properties have been established in several samples, including the United Kingdom (Goodman et al., 2000) and the United States (Bourdon et al., 2005). In the three mental health domains, each symptom is scored 0–2, with domain scores ranging from 0 to 10. Cut-points associated with a “high” or “very high” probability of having a diagnosis have been established in typically developing populations of 4–17 years of age (emotional symptoms cutoffs 5 for high,7 for very high, conduct symptoms 4 and 6, ADHD symptoms 8 and 10). The instrument has also been previously used in population studies of autism and ID (Totsika, Hastings, Emerson, Berridge, & Lancaster, 2011). In a previous report of waves 1 and 2 of the SNAP cohort, we demonstrated good domain internal consistency (Simonoff et al., 2013). For wave 3, we employed the young adult version of the SDQ, described on http://www.sdqinfo.org/Adult/, which retains the same domains but includes minor changes in wording (Brann et al., 2018). We focus on dimensional symptoms scores in the models. However, for descriptive purposes only, we estimate the proportion of participants scoring above the cut-off points associated with clinical diagnosis in epidemiological samples of typically developing youth. These should be considered impressionistic only as these cut-offs have not been validated in autistic samples, nor at this age.

#### Predictor variables

These were selected from the wave 1 assessment. Given that we are examining three mental health outcomes and the sample is of moderate size, our strategy was to test a relatively modest number of predictors, selecting those thought to be methodologically most robust for the study design and/or most meaningful for interpretation of the findings.

**Figure 1.**
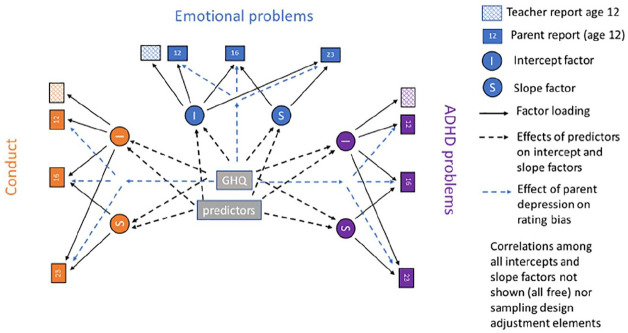
Path diagram of the multivariate growth model for SDQ conduct, emotional and ADHD at 12, 16 and 23 years of age.

1. *Child characteristics. Infant and toddler development* was retrospectively tapped using 17 items from the Diagnostic Interview for Social Communication Disorders (DISCO; Wing et al. (2002)), producing scores ranging from 0 to 34, with higher scores indicating abnormality. To provide a *language estimate* on all participants, we included the ADOS module (1, 2 or 3) used, where the selection is based on the child’s expressive language: module 1 for non-verbal children, module 2 for those with phrase speech and module 3 for fluent speakers. *Severity of autistic traits* was measured with the ADOS Calibrated Severity Score (CSS) (Gotham et al., 2012). This was selected over alternatives such as the SCQ or ADI algorithm because its measurement is independent of parent report, which forms the basis for the outcome measures. In terms of *level of functioning*, the composite score of the Vineland Adaptive Behavior Scales (VABS; (Sparrow et al. (1984)) was used as a measure of adaptive behavior. It was selected over an IQ measure because it was available on 141/158 while the broad intellectual ability of the sample had required the use of multiple cognitive tests to gain valid estimates of intellectual functioning. Among those children completing a Wechsler Intelligence Scale for Children assessment at wave 1, the VABS and WISC full-scale IQ were correlated with r = 0.57.
2. *Parental characteristics.* A binary classification of *household parental education* was scored 1 when at least one parent was educated beyond the equivalent of high school. *Parental mental health problems* at baseline were scored 1 when the informant reported any mental health problems in either parent, otherwise scored 0.
3. In relation to *contextual characteristics, neighborhood deprivation* was measured from full post codes using the Carstairs Index, which combines overcrowding, male unemployment, proportion of the population in Registrar General social class 4 and 5 (in partly skilled and unskilled employment), and households without a car; low scores represent low deprivation (Statistics, 2006). Children’s *school placement* was dichotomized as mainstream school or mainstream school with unit (coded 0) versus a special school for IDs, emotional/behavioral problems, or autism (coded 1).
4. *Informant characteristics*. In addition to possible causal associations, it is well-established in populations of typically developing (TD) children (Fergusson et al., 1993); (Kim-Cohen et al., 2005) that parental mental health may influence their perception and rating of their child’s behavior and there is emerging evidence for similar effects in populations with autism (Bennett et al., 2012; Yorke et al., 2018). Therefore, *informant affective symptoms* (where the informant was usually the mother) were measured by self-reports on the General Health Questionnaire (GHQ-30; (Goldberg & Muller, 1988)), completed by the parent providing information of their child. This was used in the model to estimate any rater effects on parental reports of their child’s mental health. In addition, teacher-reported scores on the relevant domain of the SDQ, assumed to be free of this potential parental bias, were included to anchor measurement, as shown in Figure 1.

### Statistical analysis

The repeated measures aspect of the data was addressed by the use of growth curve models, in which the correlations over time were accounted for by random effects for the intercept and, potentially, slope. Consideration of the period spanned by the three observations at ages of 12, 16 and 23, suggested simple linear growth as unlikely, so models used a log time scale from wave 1 at age 12 years in which greater change is expected during the earlier adolescent part than the later adult part of the period. That the three sub-scales of the SDQ may be correlated was accounted for by estimating the three growth curve models jointly as a single model, with the random intercepts and slopes being correlated. The random intercepts and slopes were assumed distributed as multivariate normal.

To allow us to infer that our findings related to the whole population of children with autism the models were estimated using inverse probability weights. These weights attribute greater influence to observations from the children who were relatively under-sampled/under-recruited into the study (e.g. relatively lower SCQ scores) or had a higher chance of being lost through attrition. The selective observation at wave 2 of participants who had higher IQs when first assessed was addressed by the inclusion of baseline IQ as an additional response variable correlated with all variables included in the model; the model being estimated using full-information maximum likelihood (Stata version 15, sem with method (mlmv)). This allowed both complete and incomplete observations to contribute, and to adjust for selective missingness related to variables included in the model under the assumption of missing-at-random—an assumption which includes the missing by design that related to IQ. All the analysis was undertaken in Stata version 15.

## Results

### Behavioral outcomes

Table 1 presents summary statistics for the observed parent-rated mental health outcomes, the teacher-rated wave 1 reports and the IQ variable that formed the basis of the sub-sampling at wave 2. Table 2 shows the predictor variables from wave 1 used in the modeling. The pattern of pairwise Pearson correlations depicted in Table 3, demonstrates some significant cross-sectional associations among domains, which are greater between conduct and ADHD symptoms. There is significant homotypic continuity over time, and four significant cross-domain longitudinal associations, those between wave 1 ADHD symptoms and wave 2 and wave 3 conduct symptoms, wave 2 ADHD symptoms and wave 3 emotional problems but also more unexpectedly, wave 1 emotional problems and wave 3 conduct problems.

**Table 1.** Sample characteristics—behavioral outcomes.

Variables	Wave 1 (12 years)				Wave 2 (16 years)				Wave 3 (23 years)			
	N	Mean	SD	Range	N	Mean	SD	Range	N	Mean	SD	Range
Age (years)	158	11.6	0.96	9.9–14.4	90	15.5	0.46	14.7–16.8	126	23.2	0.79	21.3–25.1
*Outcome variables*												
Parent report												
SDQ ADHD	146	7.6	2.4	0–10	84	5.9	2.5	0–10	121	5.1	2.6	0–10
SDQ Conduct	146	3.4	2.4	0–10	84	1.8	1.6	0–8	121	2.1	1.7	0–8
SDQ Emotional	146	4.6	2.7	0–10	84	3.5	2.4	0–9	121	3.9	2.4	0–9
Teacher report												
SDQ ADHD	133	4.1	1.6	0–8	–	–	–	–	–	–	–	–
SDQ Conduct	131	3.0	1.6	0–9	–	–	–	–	–	–	–	–
SDQ Emotional	132	3.5	2.5	0–10	–	–	–	–	–	–	–	–
Recorded IQ	156	72.2	24.5	19–136	–	–	–	–	–	–	–	–

SDQ: Strengths and Difficulties Questionnaire; ADHD: attention deficit hyperactivity disorder; IQ: intelligence quotient.

**Table 2.** Sample characteristics—wave 1 predictor variables.

Child characteristics	N	Mean	SD	Range
VABS	141	45.4	16.6	19–93
Behaviors in infancy (DISCO)	158	11.9	8.9	0–33
ADOS G severity score	154	6.1	2.8	
ADOS module 1/2/3, n (%)	154	22/15/ 117	(14/10 76%)	–
**Parental characteristics**	158	35	(22%)	–
Maternal GHQ	127	5.1	6.5	0–25
Parental education (% > high school diploma)	158	104	(61%)	–
Parent history of mental health problems—either parent, n (%)	145	72	(50%)	–
**Contextual characteristics**				
Carstairs neighborhood deprivation	158	−.68	2.3	−4.3 to 6.7
School placement, n (% mainstream)	158	96	(61%)	–

VABS: Vineland Adaptive Behavior Scales; DISCO: Diagnostic Interview for Social Communication Disorders; ADOS G: Autism Diagnostic Observation Schedule-Generic; GHQ: General Health Questionnaire.

**Table 3.** Pearson pairwise correlation coefficients between SDQ subscores over time (weighted).

SDQ correlations	Conduct wave 1								
Conduct wave 1	1.00	Conduct wave 2							
Conduct wave 2	0.60**	1.00	Conduct wave 3						
Conduct wave 3	0.28*	0.45*	1.00	Emotional wave 1					
Emotional wave 1	0.11	0.20	0.32*	1.00	Emotional wave 2				
Emotional wave 2	0.00	0.01	0.24	0.50**	1.00	Emotional wave 3			
Emotional wave 3	0.12	0.30	0.46**	0.43**	0.50**	1.00	ADHD wave 1		
ADHD wave 1	0.30*	0.22*	0.22*	0.09	0.12	0.07	1.00	ADHD wave 2	
ADHD wave 2	−0.28	0.08	0.16	0.18	0.15	0.24*	0.42*	1.00	ADHD wave 3
ADHD wave 3	0.03	0.22	0.50*	0.10	0.11	0.38**	0.44**	0.66**	1.00

SDQ: Strengths and Difficulties Questionnaire; ADHD: attention deficit hyperactivity disorder.

Significant results are shown in bold with *p < 0.05, **p < 0.001. Cross-domain significant results are also italicized. Coefficients that are “grouped together” (i.e. the conduct to conduct correlation coefficients, emotional to conduct correlations coefficients etc.) have been shaded.

### Model results

**Figure 2.**
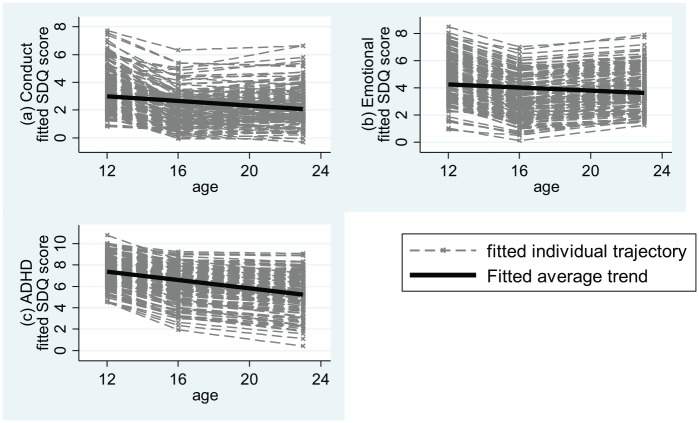
Estimated individual trajectories and average trend for SDQ conduct, emotional and ADHD scores.

The estimated growth curve model, shown in Figure 1, allowed correlation in the between-subjects variation in the baselines and change slopes for all three outcome domains. Each of the three domains of ADHD, conduct and emotional problems is allowed a separate linear growth, that estimates separate initial levels (I) and rates of increase or decrease (S) for each domain for child and allows these to be correlated over all three l domains. The model predicted individual scores at waves 1, 2, and 3 are shown in Figure 2. Model-estimated means at waves 1, 2 and 3 for conduct problems were 4.01 (95% confidence interval (CI): 3.74, 4.29), 2.31 (95% CI: 1.88, 2.74) and 2.39 (95% CI: 2.14, 2.64); for emotional problems, 4.80 (95% CI: 4.42, 5.17), 3.25 (95% CI: 2.74, 3.76) and 3.91 (95% CI: 3.57, 4.25); and for ADHD, 7.57 (95% CI: 7.30, 7.85), 6.07 (95% CI: 5.32, 6.82) and 5.35 (95% CI: 5.03, 5.66). Using the published cutoffs, the raw percentages of children in the combined high and very highly likely categories at age 12 and 23 were 50% and 37% for emotional problems, 45% and 16% for conduct problems and 62% and 21% for ADHD. As can be seen in Figure 2, the pattern of slight decline is consistent across participants.

The estimated random effects suggested no further between-subjects variation in the rate of change in problems beyond that explained by the covariates for conduct and emotional subscales, but some remained for ADHD. A linear predictor was included for each of the three baseline intercepts and their slopes—six regression-type equations. The combination of multiple linear predictors and multiple predictors implied a potentially very large number of significance tests (Supplementary Table 1). For statistical efficiency, we therefore first looked for any overall (multivariate) effect with a 6 degrees of freedom test (two parameters—intercept and slope—and three domains—emotional, conduct and ADHD symptoms) for each predictor variable and only those contributing to significant overall effects were subsequently examined. Summary statistics for the predictor variables are shown in Table 4. No overall effects were found for early infant behavior (p = 0.128), school-type (mainstream versus non-mainstream, p = 0.698), maternal GHQ (p = 0.726) and more marginally, parental history of mental health problems (p = .076). Effects of higher parental education (overall p = 0.003) arose from higher baseline emotional symptoms (p = 0.007). Effects of neighborhood deprivation as measured by the Carstairs Index (overall p = 0.002) arose from higher rates of conduct problems at baseline (p = 0.003) and a more rapid decline with age (p = .001). Greater autism symptom severity (overall p = .031) was associated with lower baseline emotional problems, while greater functional abilities as measured by the VABS (overall p = 0.006) were associated with a more rapid decline in conduct problems (p = 0.039) and ADHD symptoms (p = 0.037). Finally, lower language skills, as measured by the ADOS module (overall p = 0.014 no phrase speech versus verbally fluent, p = .001 phrases versus verbally fluent), were associated with lower levels of emotional problems at baseline (p = 0.017 and p < 0.001 respectively), and with more rapidly declining conduct problems for those with phrases (p = 0.002).

**Table 4.** Estimates of baseline risk-factor coefficients for each of the model’s intercept and slope factors for SDQ conduct, emotional and ADHD scores

	Intercept		Slope	
Baseline factor	Coefficient [95% CI]	p-value	Coefficient [95% CI]	p-value
*Outcome: SDQ conduct*				
VABS	−0.20 [−0.65, 0.25]	0.385	**−0.19** **[−0.37, −0.01]**	**0.039**
Behaviors in infancy (DISCO)	**−0.06** **[−0.11, −0.02]**	**0.010**	0.01 [−0.02, 0.03]	0.562
ADOS G severity score	−0.07 [−0.24, 0.10]	0.435	0.04 [−0.02, 0.10]	0.165
ADOS module 1 (ref category: ADOS module 3)	−1.64 [−3.42, 0.14]	0.071	−0.19 [−0.94, 0.56]	0.622
ADOS module 2 (ref category: ADOS module 3)	0.69 [−1.04, 2.41]	0.433	**−1.18** **[−1.94, −0.43]**	**0.002**
Maternal GHQ total	4.18 [−7.18, 15.55]	0.468	0.16 [−0.30, 0.62]	0.489
Parental education (ref category: no education or O-levels equivalent)	−0.19 [−1.22, 0.84]	0.710	0.42 [−0.01, 0.85]	0.054
Parent history of MH problems (Y vs N)	0.71 [−0.13, 1.56]	0.098	−0.07 [−0.41, 0.27]	0.692
Carstairs deprivation	**0.35** **[0.12, 0.58]**	**0.003**	**−0.14** **[−0.23, −0.06]**	**0.001**
School (non-mainstream vs mainstream)	0.39 [−0.85, 1.63]	0.538	−0.14 [−0.62, 0.34]	0.564
*Outcome: SDQ emotional*				
VABS	−0.27 [−0.72, 0.17]	0.230	−0.02 [−0.27, 0.22]	0.852
Behaviors in infancy (DISCO)	−0.00 [−0.05, 0.05]	0.958	0.01 [−0.02, 0.03]	0.588
ADOS G severity score	**−0.15** **[−0.29, −0.01]**	**0.030**	0.01 [−0.05, 0.08]	0.670
ADOS module 1 (ref category: ADOS module 3)	**−2.27** **[−4.12, −0.41]**	**0.017**	0.46 [−0.46, 1.37]	0.329
ADOS module 2 (ref category: ADOS module 3)	**−2.52** **[−3.86, −1.17]**	**<** **0.001**	0.29 [−0.42, 0.99]	0.420
Maternal GHQ total	0.21 [−2.31, 2.73]	0.868	0.16 [−0.40, 0.73]	0.570
Parental education (ref category: no education or O-levels equivalent)	**1.40** **[0.38, 2.41]**	**0.007**	−0.30 [−0.78, 0.18]	0.218
Parent history of MH problems (Y vs N)	0.76 [−0.10, 1.62]	0.083	−0.02 [−0.49, 0.45]	0.938
Carstairs deprivation	0.16 [−0.05, 0.36]	0.131	−0.06 [−0.17, 0.04]	0.256
School (non-mainstream vs mainstream)	0.16 [−0.63, 0.95]	0.684	0.05 [−0.40, 0.50]	0.826
*Outcome: SDQ hyperactivity*				
VABS	−0.43 [−0.89, 0.03]	0.065	**−0.27** **[−0.52, −0.02]**	**0.037**
Behaviors in infancy (DISCO)	0.01 [−0.04, 0.05]	0.799	−0.01 [−0.04, 0.02]	0.453
ADOS G severity score	0.10 [−0.10, 0.29]	0.348	−0.06 [−0.16, 0.04]	0.230
ADOS module 1 (ref category: ADOS module 3)	−1.63 [−3.62, 0.37]	0.109	−0.17 [−0.94, 0.59]	0.654
ADOS module 2 (ref category: ADOS module 3)	0.55 [−0.52, 1.63]	0.310	−0.78 [−1.64, 0.07]	0.073
Maternal GHQ total	−0.41 [−1.92, 1.09]	0.587	0.12 [−0.45, 0.68]	0.683
Parental education (ref category: No education or O-levels equivalent)	0.79 [−0.05, 1.63]	0.065	0.25 [−0.26, 0.76]	0.341
Parent history of MH problems (Y vs N)	−0.43 [−1.27, 0.40]	0.307	0.07 [−0.39, 0.53]	0.770
Carstairs deprivation	0.03 [−0.14, 0.20]	0.756	0.05 [−0.05, 0.14]	0.341
School (non-mainstream vs mainstream)	−0.29 [−1.11, 0.52]	0.479	−0.15 [−0.80, 0.50]	0.641

SDQ: Strengths and Difficulties Questionnaire; ADHD: attention deficit hyperactivity disorder; CI: confidence interval; VABS: Vineland Adaptive Behavior Scales; DISCO: Diagnostic Interview for Social Communication Disorders; ADOS G: Autism Diagnostic Observation Schedule-Generic; GHQ: General Health Questionnaire; MH: mental health.

Results for those predictor variables surviving overall tests of effects are presented, with bivariate significance levels. No significant depression-associated effect was found for the parental ratings in any of the three domains.

## Discussion

### Overall pattern of trajectories

The present study examines the trajectories of emotional, conduct and ADHD symptoms over 11 years in a population-based sample of participants with autism. Using the same measure at three timepoints, we demonstrate small but significant declines in symptoms in all three domains of psychopathology. However, despite these declines, a high proportion of participants retain scores above the reported cutoffs, especially for emotional problems and ADHD. While these cutoffs should be viewed with caution as the SDQ is a screening instrument and has not been validated for autistic populations or for those over 18 years, these high rates nevertheless support the importance of considering mental health at all ages in people with autism. The finding of elevated levels of mental health symptoms concurs with the available literature on high prevalence rates of psychiatric disorder across the age range from childhood to adult life, which is striking in the consistency of reporting high rates of mental health symptoms and disorders (Croen et al., 2015; Gjevik et al., 2011; Helles et al., 2017; Lai et al., 2019; Lever & Geurts, 2016; Lugo-Marín et al., 2019; Rydzewska et al., 2018; Salazar et al., 2015; Simonoff et al., 2008).

The decline in mental health symptoms over time is in line with several previous studies measuring predominantly behavioral problems (Anderson et al., 2011; Gray et al., 2012; Shattuck et al., 2007). These studies differ from SNAP in relation to age (both having greater within-sample variation and measuring change over different age periods, child to adolescent or late adolescence to mid-adulthood) and also in terms of the measures of psychopathology, as these other studies have a greater focus on ‘maladaptive‘ behaviors. However, one study, the EDX cohort which tracked trajectories from 13 to 24 years, differs from SNAP and the other cohorts with respect to anxiety and depression trajectories, reporting increases across adolescence into early adulthood, especially in females (Gotham et al., 2015). While EDX differs from previously reported studies with respect to age, there is considerable age overlap with SNAP. Whether this difference is due to measurement is not clear. EDX employed the child and adult versions of the Child Behavior Checklist (CBCL; (Achenbach & Rescorla, 2006)). In typically developing populations, the CBCL and the SDQ produce similar results (Goodman & Scott, 1999). However, the SDQ is considerably shorter (25 vs 113 items) and differences in type and extent of item content could be important in atypical populations such as autism. Of note, Gotham et al. (2015) found age by sex interactions with the greater increase in anxiety and depression on the Achenbach scales in females, thus highlighting an important potential stratification for future research, as males predominate in most existing cohorts. The present analyses on SNAP did not explore sex differences due to the small number of females.

In line with its population-based derivation, the SNAP cohort is heterogeneous in terms of autism symptoms severity (ADOS severity score range 1–10) and adaptive function (Vineland scores 19–93). Furthermore, wave 1 mental health symptom scores for each domain spanned the entire range (0–10). Despite this extensive variation within the cohort, the latent growth curve models, which took account of baseline score as well as a range of predictor variables, revealed high levels of stability and accounted well for the individual differences, especially for emotional and behavioral symptoms, and did not suggest that there were other important sources of heterogeneity in trajectory prediction.

### Predictors of individual trajectories

#### Conduct symptoms

In comparing the present findings to previous reports, the SDQ conduct domain used here maps to the Developmental Behaviour Checklist (DBC) disruptive domain used by Gray et al. (2012), and somewhat to both the irritability subscale of the Applied Behavioral Analysis (ABC) employed by Anderson et al. (2011) and the externalizing scale of the Scales of Independent Behavior—Revised (SIB-R; (Bruininks et al., 1996)) used in the Adolescents and Adults with Autism (AAA) study (Seltzer et al., 2003). Higher level of functioning predicted a greater reduction in externalizing problems whether measured by IQ (Anderson et al., 2011) or presence of an ID (Shattuck et al., 2007) in most studies but not all (Gray et al., 2012). Consistent with others measuring IQ, we found that a higher adaptive function as measured on the VABS score was associated with a greater decline in conduct problems, supporting previous findings. Furthermore, we also found that lower language level at baseline (ADOS module 2 versus 3) predicted less decline in conduct problems, lending robustness to the finding but also highlighting that the exact mechanism of effect needs elucidation. It is well recognized that lower verbal skills are linked to greater rates of externalizing behavior in developmental disorders (McClintock et al., 2003). It will be important that future research attempts to distinguish if this effect can be substantially ameliorated by providing alternative, non-verbal means of communication or whether it reflects underpinning factors associated with lower global cognitive ability. In examining language level at an older age, the AAA study did not find a relationship to externalizing behaviors (Shattuck et al., 2007), raising the possibility the effect is more time-limited. Interestingly, we failed to identify a relationship of either baseline level or change in conduct symptoms to severity of autism as measured on the ADOS, suggesting the risk factor for this domain may be communication rather than social communication.

Previously, we reported in the SNAP cohort that higher levels of neighborhood deprivation were associated with a greater reduction in conduct symptoms between wave 1 and wave 2. Here, we confirm the earlier finding with the addition of data from wave 3. Furthermore, the statistically efficient analysis undertaken here also identified deprivation as being associated with a higher level of baseline conduct symptoms. Our finding is consistent with research in typically developing populations, where indices of deprivation are strongly associated with greater levels of conduct problems, although the effect may become non-significant in multivariate analyses that include other metrics associated with psychosocial disadvantage, raising questions about the mechanism of effect and whether neighborhood deprivation has a direct effect (e.g. Ford et al., 2004). The previous literature with respect to autism is limited. Gray et al. (2012) did not find a relationship of antisocial symptoms to socioeconomic disadvantage in their Australian sample. In a younger Australian cohort, Emerson reported that environmental risk factors, including neighborhood deprivation, increased risk of conduct problems in 3-year-olds with ASD similarly to that in typically developing and intellectually disabled comparison groups, and also that these risk factors led to greater persistence in autistic children at 5 and 7 years, compared to the other groups (Emerson et al., 2014). This contrasts with our finding that neighborhood deprivation was associated with greater decline in conduct symptoms over time, albeit at a different timepoint in development. The analyses by Emerson et al. (2014) were bivariate and did not account for multivariate indices of environmental risk, making it difficult to infer a direct effect of neighborhood deprivation. Neighborhood deprivation is associated with a wide range of potential risk factors for mental health problems, including poverty, parental mental and physical ill-health, lack of social cohesion and opportunities for community participation (Fone et al., 2014) and meta-analysis has shown that accounting for these variables can alter the relationship between deprivation and other outcomes (Nieuwenhuis & Hooimeijer, 2016). In terms of our present findings, the effect of neighborhood deprivation was identified in multivariate analyses, which included correlated factors of parental mental health and parental education but no other wider environmental correlates of neighborhood deprivation. Future research should aim to replicate our finding and more precisely identify the more proximal risk factors driving this relationship.

We found no significant effect of parental mental health on any domains, whether measured historically or at baseline. In relation to externalizing symptoms, the AAA study identified that a better mother–child relationship was linked to lower baseline and maternal praise predicted a greater decline in problems (Woodman et al., 2015). As poorer parental mental health is associated with parenting stress and parent–child relationships in typically developing samples (Deater-Deckard, 2014) and parents of autistic children often experience higher levels of stress and mental ill-health (Hastings et al., 2005; Yorke et al., 2018), future longitudinal studies should explore this area in more detail

#### Emotional symptoms

We found that both greater autism severity and lower language level were associated with lower baseline parent-reported emotional symptoms. Gotham et al. (2015) found that higher verbal IQ was associated with greater anxiety but not depression scores and Shattuck et al. (2007) found that those with ID had less decline in internalizing symptoms. We include adaptive function as measured on the VABS composite, rather than IQ which was measured on a variety of measures, in the current study. Although these measures are strongly related (r = 0.57 in this study), they are nevertheless different phenomena, with the VABS composite indexing a much wider range of performance. However, these differences also mirror a large body of inconsistent research on the cross-sectional relationship between IQ and levels of affective symptoms in autism. The literature reports both that anxiety is related to higher IQ (Gadow et al., 2005; Hallett et al., 2013; Weisbrot et al., 2005) or is not different according to IQ (Brereton et al., 2006; de Bruin et al., 2007; Simonoff et al., 2008). Furthermore, the role of IQ may vary across subdomains of anxiety (Witwer & Lecavalier, 2008). The reporting of affective symptoms, requiring inference of another’s internal experiences, may be particularly challenging in autism, where impairments in communication and emotional literacy are common. The style of assessment (interview versus questionnaires) and the content may play a greater role in accurately identifying psychopathology. This uncertainty highlights the pressing need to validate measures of mental health symptoms for autistic people.

In relation to family characteristics, we found that higher parental education was associated with reports of greater emotional problems. The AAA study did not show such a link (Woodman et al., 2016) but Gotham et al. (2015) found that mothers with less education reported greater increases in depressive symptoms in the subsample with lower verbal ability. Again, whether these differences reflect true symptomatic variation or biases in measurement needs evaluation in future studies. The findings from Gotham et al. (2015) with respect to females are intriguing. Very few samples, including SNAP, have sufficient numbers of females to test robustly for sex differences, and this should be a recruitment priority for future cohorts.

#### ADHD symptoms

We found that higher VABS scores at wave 1 predicted a greater decline in ADHD symptoms, but no other relationships met our tests of significance. Previously in this sample, when not accounting for multiple testing, we reported that wave 1 higher IQ and higher VABS both predicted lower wave 2 absolute scores but not change (Simonoff et al., 2013). Consistent with our present findings, Anderson et al. (2011) reported that higher verbal IQ predicted a greater decline in symptoms of hyperactivity on the ABC.

#### Other predictors

Of note, we failed to find any significant prediction for a history of parental mental health problems (trend association with emotional symptoms intercept = 0.083) or for school placement. Parental mental health problems is a robust predictor of childhood symptoms in general population studies (Ford et al., 2004) and parents of autistic children appear to be at greater risk, probably both for genetic (Bolton et al., 1998; Piven & Palmer, 1999) and likely environmental reasons, manifesting as parental stress (Estes et al., 2009). Hence, it is particularly striking that there was very little evidence for a role of such difficulties in the level and persistence of mental health symptoms in their offspring. It should be borne in mind that, like all our measures, a history of parental mental health problems was captured at baseline and we did not include measures at later timepoints, as these make interpretation of trajectories more difficult In addition to this direct role, we tested for a possible informant rater effect, as our outcome measures were solely reported by parents. Reassuringly, we failed to find evidence supporting a rater effect suggesting bias due to parental affective symptoms.

The null finding with respect to school placement is of particular interest as we have found in the SNAP cohort that exposure to mainstream schooling predicts a relative decline in autistic symptoms over the same time period (Simonoff et al., 2019). These previous analyses did not examine collateral effects of different schooling experiences and a concern was that mainstream school attendance might increase levels of emotional symptoms, in particular, anxiety in relation to greater actual or perceived performance demands. The present findings do not support this potential concern, although it should be noted that the analytic strategy did not attempt to partial out effects between waves 1 and 2 (the school-age period) and waves 2 and 3. Our earlier report of wave 1 to 2 relationships similarly failed to identify a role for school placement in any of the symptom domains, supporting the present results (Simonoff et al., 2013).

The present study has several strengths. The sample is population-representative, and the use of inverse probability weights means that the estimates reflect the wider population of people with autism from which the sample was drawn. We had high retention levels at wave 3, and the analytic procedure took account of any selective attrition, including the purposive sampling at wave 2, so that estimates are unbiased. In order to avoid false positive results, we employed an efficient analysis strategy of a single model with parsimonious selection of predictor measures, which should increase confidence in the positive findings. We were able, however, to include a broader range of potential predictor variables than that of any previous longitudinal analysis. To increase clarity of interpretation, we aimed to use predictor variables that would not share method variance with the outcome, for example, the ADOS CSS for autism severity, and we accounted for possible rater effects by including baseline teacher ratings on the SDQ and the maternal GHQ score.

An important limitation of the study is the use of the SDQ subscales to measure mental health domains. While a well-recognized and validated measure for research studies in non-autistic populations, like the overwhelming majority of currently available measures, its sensitivity and accuracy in autism are uncertain. As mental health symptoms may have atypical manifestation in autism (Kerns & Kendall, 2012), brief questionnaires may potentially miss important psychopathology. This issue of valid psychopathology measurement in autism needs urgent attention. The SNAP cohort has a limited number of female participants, and we judged there was insufficient power to explore sex differences. However, sex difference in the mental health of autistic people is a particularly important research priority for future studies. The SNAP cohort was established before there was a general appreciation of the importance of mental health conditions in autism, and the early assessments were not designed to measure mental health risk factors. Only variables collected at wave 1 are used as predictors, as the interpretation of the predictive role of subsequently collected data would be unclear. However, this places a limitation on the interpretation of factors that may change over time, such as parental mental health and neighborhood characteristics. We did not obtain systematic information about any interventions received across the entire study period, and it is therefore not possible to take account of how treatment may have influenced individual trajectories. But, even if they had been measured their heterogeneity in terms of type, intensity, duration, and timing would have made estimating any causal effect challenging. Finally, we should bear in mind the modest sample size, increasing likely inferential error rates and the need for replication of findings in other cohorts.

In summary, we report the mental health trajectories in a population-based cohort from middle childhood to adult life for the three common domains of psychopathology. We show that while symptoms in all domains decline over this time period, a substantial proportion still remain above the currently quoted cutoffs for a likely disorder at least one area. The striking consistency in rank order over time lends hope to the expectation that in the near future it should be possible to identify early which individuals will be at the greatest risk for persisting mental health problems. There were few predictors of trajectories across domains, but better adaptive functioning on the VABS predicted a more rapid decrease in both conduct and ADHD symptoms and higher initial language level predicted greater decline in conduct and, at trend level, ADHD symptoms. Interestingly, better initial language also predicted higher emotional symptoms and it is unclear whether this relationship is true or artefactual. Both language and adaptive function are frequent targets for intervention in autism and future research should evaluate the effect of autism-focused interventions on co-occurring psychopathology. Given the prevalence and persistence of additional mental health problems in autism, a further research priority will be to test the role of theory-driven risk factors.

## Supplemental Material

SDQ_trajectories_Supplementary_materials – Supplemental material for Trajectories of emotional and behavioral problems from childhood to early adult life Click here for additional data file. Supplemental material, SDQ_trajectories_Supplementary_materials for Trajectories of emotional and behavioral problems from childhood to early adult life by Dominic Stringer, Rachel Kent, Jackie Briskman, Steve Lukito, Tony Charman, Gillian Baird, Catherine Lord, Andrew Pickles and Emily Simonoff in Autism
