# Supplementary material for: Trajectories of emotional and behavioral problems from childhood to early adult life
Source: Autism. 2020 Mar 19;24(4):1011–24. doi: 10.1177/1362361320908972 (PMC7521012; doi:10.1177/1362361320908972)
Supplement: SDQ_trajectories_Supplementary_materials – Supplemental material for Trajectories of emotional and behavioral problems from childhood to early adult life [file SDQ_trajectories_Supplementary_materials.pdf]

Supplementary Figure 1

SNAP cohort participation over waves 1-3

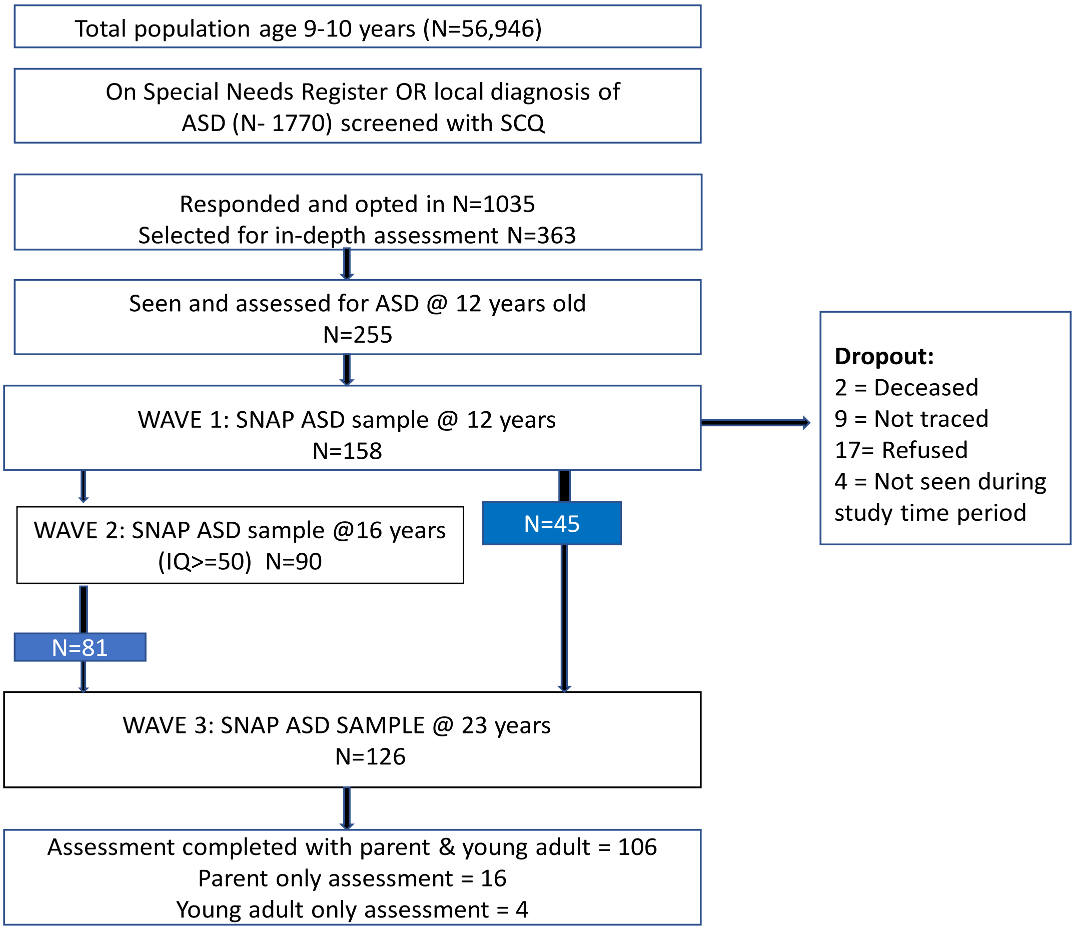

Supplementary Table 1 – Overall tests of predictors

	p-value
School (non-mainstream vs mainstream)	0.698
Parental education<br><br>(ref category: No education or O-levels equivalent)	**0.003**
Carstairs deprivation	**0.002**
ADOS G Severity Score	**0.031**
Behaviours in infancy (DISCO)	0.128
VABS	**0.006**
ADOS module 1 (ref category: ADOS module 3)	**0.014**
ADOS Module 2 (ref category: ADOS module 3)	**0.001**
Maternal GHQ total	0.726
Parent history of MH problems (Y vs N)	0.076
